# Supplementary material for: Microtubule plus-end dynamics link wound repair to the innate immune response
Source: eLife. 2020 Jan 29;9:e45047. doi: 10.7554/eLife.45047 (PMC7043892; doi:10.7554/eLife.45047)
Supplement: Figure 2—source data 1. [file elife-45047-fig2-data1.docx]

# Table 1

Reporter protein dynamics in the epidermis

| **Type of Dynamics** | **Transgene** | **Velocity (mean ± SEM) μm s^−1^** | |
| --- | --- | --- | --- |
|  |  | before wound | after wound |
| MT plus-end growth | *col-19p::EBP-2::GFP* | 0.26 ± 0.10 µm/sec  (n=33) | 0.16 ± 0.06 µm/sec  (n=33) |
| endosome transport | *dpy-7p::GFP::RAB-11* | 1.31 ± 0.33 µm/sec  (n=50) | 1.14 ± 0.47 µm/sec  (n=40) |
| SNF-12 | *col-12p::SNF-12::GFP* | immobile* | 0.007 ± 0.004 µm/sec  (n=40) |

*less than 1% of SNF-12 clusters move with a speed of 0.017 ± 0.005 µm/sec (n=21)
