# Supplementary material for: Microtubule plus-end dynamics link wound repair to the innate immune response
Source: eLife. 2020 Jan 29;9:e45047. doi: 10.7554/eLife.45047 (PMC7043892; doi:10.7554/eLife.45047)
Supplement: Supplementary file 1. [file elife-45047-supp1.docx]

## Table S1. MT-related genes identified in a genome-wide screen for regulators of AMP gene expression from (Zugasti et al., 2016).

| **WormBase Gene ID (WS266)** | **Public Name** | **Sequence Name** | **Description** |
| --- | --- | --- | --- |
| WBGene00000962 | *dhc-1* | T21E12.4 | dynein heavy chain homolog |
| WBGene00001017 | *dnc-1* | ZK593.5 | dynactin complex subunit p150 homolog |
| WBGene00001832 | *hcp-4* | T03F1.9 | centromere protein (CENP)-C homolog |
| WBGene00002231 | *knl-1* | C02F5.1 | essential kinetochore component |
| WBGene00002845 | *let-711* | F57B9.2 | NOT1 orthologue |
| WBGene00004274 | *rab-11.1* | F53G12.1 | small GTPase homologous to the Rab GTPases |
| WBGene00010556 | *rack-1* | K04D7.1 | orthologue of vertebrate Receptor for Activated C Kinase |
| WBGene00006529 | *tba-2* | C47B2.3 | alpha-tubulin |
| WBGene00006530 | *tba-4* | F44F4.11 | alpha tubulin |

##

Zugasti, O., Thakur, N., Belougne, J., Squiban, B., Kurz, C.L., Soule, J., Omi, S., Tichit, L., Pujol, N., and Ewbank, J.J. (2016). A quantitative genome-wide RNAi screen in *C. elegans* for antifungal innate immunity genes. BMC Biol *14*, 35.
