# Supplementary material for: Microtubule plus-end dynamics link wound repair to the innate immune response
Source: eLife. 2020 Jan 29;9:e45047. doi: 10.7554/eLife.45047 (PMC7043892; doi:10.7554/eLife.45047)
Supplement: Supplementary file 2. [file elife-45047-supp2.pdf]

**Key Resources Table**				
**Reagent type (species) or resource**	**Designation**	**Source or reference**	**Identifiers**	**Additional information**
strain, strain background (*Escherichia coli*)	*E. coli*: OP50	*Caenorhabditis* Genetics Center	RRID: WB-STRAIN:OP50	
strain, strain background (*Escherichia coli*)	Ahringer RNAi library	(Kamath and Ahringer 2003)		
strain, strain background (*Escherichia coli*)	Vidal RNAi library	(Rual, et al. 2004)		
strain, strain background (*fungi*)	*Drechmeria coniospora*	(Lebrigand, et al. 2016)		
Genetic reagent (*C. elegans*)	IG274: *frIs7[nlp-29p::GFP, col-12p::DsRed] IV*	(Pujol, et al. 2008)		
Genetic reagent (*C. elegans*)	IG823: *frIs43[col-12p::SNF-12::GFP, ttx-3p::DsRed2] V*	(Dierking, et al. 2011)		
Genetic reagent (*C. elegans*)	IG1235: *cdIs73[RME-8::mRFP, unc-119(+); ttx-3p::GFP]; frIs43[col-12p::SNF-12::GFP, ttx-3p::DsRed2]*	this study		Materials and methods section
Genetic reagent (*C. elegans*)	IG1236: *pwIs82[snx-1p::mRFP::SNX-1, unc-119(+)]; frIs43[col-12p::SNF- 12::GFP, ttx-3p::DsRed2]*	this study		Materials and methods section

Genetic reagent (*C. elegans*)	IG1270: *frEx453[pMS8(col-12p::GFP::STA-2),pMS9(col-12p::mCherry::SNF-12)]*	this study		Materials and methods section
Genetic reagent (*C. elegans*)	IG1623: *frSi9[pNP151(col-62p::Lifeact::mKate2_3'c-nmy), unc-119(+) ttTi5605] II*	this study		Materials and methods section
Genetic reagent (*C. elegans*)	IG1659: *frSi13[pNP159(dpy-7p::GFP::RAB-11 ttTi5605)] II*	this study		Materials and methods section
Genetic reagent (*C. elegans*)	IG1663: *frEx577[pNP158(snf-12p::SNF-12::GFP_3'snf-12, pCFJ90(myo-2p::mCherry), pCFJ104(myo-3p::mCherry)]*	this study		Materials and methods section
Genetic reagent (*C. elegans*)	IG1675: *frSi9[pNP151(col-62p::Lifeact::mKate2_ 3'c-nmy), unc-119(+) ttTi5605] II; juEx3762[col-19p::EBP-2::GFP, ttx-3p::RFP]*	this study		Materials and methods section
Genetic reagent (*C. elegans*)	IG1677: *frSi9[pNP151(col-62p::Lifeact::mKate2_3'c-nmy), unc-119(+) ttTi5605] II; frEx314[col-12p::SNF-12::GFP, ttx-3p::DsRed2]*	this study		Materials and methods section
Genetic reagent (*C. elegans*)	IG1720: *+/mcIs54[dpy-7p::SPAS-1_IRES_NLSmCherry, unc-119(+)] X; frEx314[col-12p::SNF-12::GFP, ttx-3p::DsRed2]*	this study		Materials and methods section
Genetic reagent (*C. elegans*)	IG1723: *+/mcIs54[dpy-7p::SPAS-1_IRES_NLSmCherry , unc-119(+)] X; juSi239[col-*	this study		Materials and methods section

	19p::GFP::TBB-2] I; frEx314[col-12p::SNF-12::GFP, ttx-3p::DsRed2]			
Genetic reagent (*C. elegans*)	IG1726: *mcIs54[dpy-7p::SPAS-1_IRES_NLSmCherry, unc-119(+)] X; juEx3762[col-19p::EBP-2::GFP, ttx-3p::RFP]*	this study		Materials and methods section
Genetic reagent (*C. elegans*)	IG1729: *frSi16[pSO10(SEC::snf-12p::SNF-12::EGFP_3'snf-12) ttTi4348] I*	this study		Materials and methods section
Genetic reagent (*C. elegans*)	IG1784: *frEx597[pSO16(col-12p::SNF-12::mKate2_3'unc-54), pCFJ90(myo-2p::mCherry), pKS]*	this study		Materials and methods section
Genetic reagent (*C. elegans*)	IG1792: *frSi13[pNP158(dpy-7p::GFP::RAB-11) ttTi5605] II; frEx597[pSO16(col-12p::SNF-12::mKate2_3'unc-54), pCFJ90(myo-2p::mCherry), pKS]*	this study		Materials and methods section
Genetic reagent (*C. elegans*)	IG1796: *frIs43[col-12p::SNF-12::GFP, ttx-3p::DsRed2] V; qxIs352[ced-1p::LAAT-1::mCherry]*	this study		Materials and methods section
Genetic reagent (*C. elegans*)	IG1798: *frIs43[col-12p::SNF-12::GFP, ttx-3p::DsRed2] V; qxIs68[ced-1p::mCherry::RAB-7]*	this study		Materials and methods section
Genetic reagent (*C. elegans*)	IG1799: *frIs43[col-12p::SNF-12::GFP, ttx-3p::DsRed2] V; qxIs513[ced-1p::mCherry::PLC-1-PH]*	this study		Materials and methods section

Genetic reagent (*C. elegans*)	IG1813: *frSi9[pNP151(col-62p::Lifeact::mKate2_3'c-nmy), unc-119(+) ttTi5605]/+ II; tbb-2(tj26[GFP::TBB-2]) III*	this study		Materials and methods section
Genetic reagent (*C. elegans*)	IG1848: *ebp-2(wow47[EBP-2::GFP]) II*	this study		Materials and methods section
Genetic reagent (*C. elegans*)	IG1933: *frSi9[pNP151(col-62p::Lifeact::mKate2_3'c-nmy), unc-119(+) ttTi5605] II; dnc-2[prt42(3xflag::gfp::DHC-2)] III*	this study		Materials and methods section
Genetic reagent (*C. elegans*)	IG1932: *maph-1.1(mib12[GFP::MAPH-1.1]) I; frSi9[pNP151(col-62p::Lifeact::mKate2_3'c-nmy), unc-119(+) ttTi5605] II*	this study		Materials and methods section
Genetic reagent (*C. elegans*)	IG1936: *tba-1(pg77[TagRFP-T::TBA-1]) I; frSi9[pNP151(col-62p::Lifeact::mKate2_3'c-nmy), unc-119(+) ttTi5605] II; juEx3762[col-19p::EBP-2::GFP, ttx-3p::RFP]*	this study		Materials and methods section
Genetic reagent (*C. elegans*)	IG1327: *rde-1(ne219) V; juIs346[col-19p::RDE-1, ttx-3p::GFP] III; frIs7[nlp-29p::GFP, col-12p::DsRed] IV*	(Zugasti, et al. 2014)		RNAi sensitive in the adult epidermis
Genetic reagent (*C. elegans*)	CZ13896: *juls319[col-19p::GCaMP3, col-19p::tdTomato]*	(Xu and Chisholm 2011)		
Genetic reagent (*C. elegans*)	CZ14453: *juEx3762[col-19p::EBP-2::GFP, ttx-3p::RFP]*	(Chuang, et al. 2016)		

Genetic reagent (*C. elegans*)	CZ14748: *juls352[GFP::moesin, ttx-3p::RFP] I*	(Xu and Chisholm 2011)		
Genetic reagent (*C. elegans*)	CZ21789: *juSi239[col-19p::GFP::TBB-2] I*	(Chuang, et al. 2016)		
Genetic reagent (*C. elegans*)	CZ9334: *juEx1919[dpy-7p::GFP::RAB-5, ttx-3p::RFP]*	(Chuang, et al. 2016)		
Genetic reagent (*C. elegans*)	MBA365: *Ex[dpy-7p::GFP::CAAX_3'unc-54, myo-2p::GFP]*	Michalis Barkoulas, UCL		
Genetic reagent (*C. elegans*)	ML1896: *mcIs35[lin-26p::GFP::TBA-2, pat-4p::CFP, rol-6(su1006)]; mcIs54[dpy- 7p::SPAS-1_IRES_NLSmCherry, unc-119(+)] X*	(Quintin, et al. 2016)		
Genetic reagent (*C. elegans*)	NP878: *unc-119(ed3) III; cdIs73[RME-8::mRFP, ttx-3p::GFP, unc-119(+)]*	(Shi, et al. 2009)		
Genetic reagent (*C. elegans*)	RT343: *pwIs82[snx-1p::mRFP::SNX-1, unc-119(+)]*	(Sato, et al. 2014)		
Genetic reagent (*C. elegans*)	SA854: *tbb-2(tj26[GFP::TBB-2]) III*	(Honda, et al. 2017)		
Genetic reagent (*C. elegans*)	SA884: *tbb-1(tj30[GFP::TBB-1]) III*	(Honda, et al. 2017)		
Genetic reagent (*C. elegans*)	SV1009: *Is[wrt-2p::GFP::PH-PLC1δ, wrt-2p::GFP::H2B, lin-48p::mCherry]*	(Wildwater, et al. 2011)		

Genetic reagent (*C. elegans*)	XW6096: *qxIs352[ced-1p::LAAT-1::mCherry]*	(Liu, et al. 2012)		
Genetic reagent (*C. elegans*)	XW9653: *qxIs68[ced-1p::mCherry::RAB-7]*	(Liu, et al. 2012)		
Genetic reagent (*C. elegans*)	XW10992: *qxIs513[ced-1p::mCherry::PLC-1-PH]*	(Liu, et al. 2012)		
Genetic reagent (*C. elegans*)	JLF302: *ebp-2(wow47[EBP-2::GFP]) II; zif-1(gk117) III*	(Sallee, et al. 2018)		
Genetic reagent (*C. elegans*)	GOU2047 : arx-2(cas607[GFP::ARX-2])	(Wu, et al. 2017)		
Genetic reagent (*C. elegans*)	BOX188*:* *maph-1.1(mib12[GFP::MAPH-1.1]) I*	(Waaijers, et al. 2016)		
Genetic reagent (*C. elegans*)	GCP417 : *dnc-2[prt42(3xflag::gfp::DHC-2)] III*	(Barbosa, et al. 2017)		
Genetic reagent (*C. elegans*)	GN675: *tba-1(pg77[TagRFP-T::TBA-1]) I*	(Lockhead, et al. 2016)		
Recombinant DNA reagent	Plasmid *pSO16 (col-12p::SNF-12::mKate2_3’unc-54)*	This study		Materials and methods section
Recombinant DNA reagent	Plasmid *pSO10 (SEC::snf-12p::SNF-12::EGFP_3'snf-12)*	This study		Materials and methods section

Recombinant DNA reagent	Plasmid pNP159 (*dpy-7p::GFP::RAB-11*)	This study		RAB-11 reporter in the epidermis
Recombinant DNA reagent	Plasmid pNP158 *(snf-12p::SNF-12::GFP_3'snf-12)*	This study		Materials and methods section
Recombinant DNA reagent	Plasmid pNP151 *(col-62p::Lifeact::mKate2_3'c-nmy)*	This study		Actin reporter in the adult epidermis
Chemical compound, drug	levamisole	ACROS Organics™		
Commercial assay or kit	Gibson Assembly<br>Image NIH imageJ RRID:SCR_003070<br>J	New England Biolabs		
Software, algorithm	Fiji/ImageJ	NIH image	RRID: SCR_003070	
Software, algorithm	ZEN	Zeiss https://www.zeiss.com/microscopy/us/downloads/zen.html		
Software, algorithm	VisiView	Visitron https://www.visitron.de/products/visiviewr-software.html		

Software, algorithm	Metamorph	https://www.moleculardevices.com/products/cellular-imaging-systems/acquisition-and-analysis-software/metamorph-microscopy#gref		
Software, algorithm	R	https://www.r-project.org/		
Software, algorithm	Matlab	https://www.mathworks.com/products/matlab.html		
Software, algorithm	GraphPad Prism 5	GraphPad Software, Inc.	RRID: SCR_002798	
Software, algorithm	CloneMapper	(Thakur, et al. 2014)		

Barbosa, D. J., et al.
2017 Dynactin binding to tyrosinated microtubules promotes centrosome centration in *C. elegans* by enhancing dynein-mediated organelle transport. PLoS Genet 13(7):e1006941.
Chuang, M., et al.
2016 DAPK interacts with Patronin and the microtubule cytoskeleton in epidermal development and wound repair. Elife 5.
Dierking, K., et al.
2011 Unusual regulation of a STAT protein by an SLC6 family transporter in *C. elegans* epidermal innate immunity. Cell Host Microbe 9(5):425-35.
Honda, Y., et al.

2017 Tubulin isotype substitution revealed that isotype combination modulates microtubule dynamics in *C. elegans* embryos. J Cell Sci 130(9):1652-1661.
Kamath, R. S., and J. Ahringer
2003 Genome-wide RNAi screening in *Caenorhabditis elegans*. Methods 30(4):313-21.
Lebrigand, K., et al.
2016 Comparative Genomic Analysis of *Drechmeria coniospora* Reveals Core and Specific Genetic Requirements for Fungal Endoparasitism of Nematodes. PLoS Genet 12(5):e1006017.
Liu, B., et al.
2012 LAAT-1 is the lysosomal lysine/arginine transporter that maintains amino acid homeostasis. Science 337(6092):351-4.
Lockhead, D., et al.
2016 The tubulin repertoire of *C. elegans* sensory neurons and its context-dependent role in process outgrowth. Mol Biol Cell.
Pujol, N., et al.
2008 Distinct innate immune responses to infection and wounding in the *C. elegans* epidermis. Curr Biol 18(7):481-9.
Quintin, S., et al.
2016 Non-centrosomal epidermal microtubules act in parallel to LET-502/ROCK to promote *C. elegans* elongation. Development 143(1):160-73.
Rual, J. F., et al.
2004 Toward improving *Caenorhabditis elegans* phenome mapping with an ORFeome-based RNAi library. Genome Res 14(10B):2162-8.
Sallee, M. D., et al.
2018 Tissue-specific degradation of essential centrosome components reveals distinct microtubule populations at microtubule organizing centers. PLoS Biol 16(8):e2005189.
Sato, K., et al.
2014 *C. elegans* as a model for membrane traffic. WormBook:1-47.
Shi, A., et al.
2009 Regulation of endosomal clathrin and retromer-mediated endosome to Golgi retrograde transport by the J-domain protein RME-8. EMBO J 28(21):3290-302.
Thakur, N., et al.
2014 Clone mapper: an online suite of tools for RNAi experiments in *Caenorhabditis elegans*. G3 4(11):2137-45.
Waaijers, S., et al.
2016 A tissue-specific protein purification approach in *Caenorhabditis elegans* identifies novel interaction partners of DLG-1/Discs large. BMC Biol 14:66.
Wildwater, M., et al.
2011 Cell shape and Wnt signaling redundantly control the division axis of *C. elegans* epithelial stem cells. Development 138(20):4375-85.
Wu, D., et al.
2017 CED-10-WASP-Arp2/3 signaling axis regulates apoptotic cell corpse engulfment in *C. elegans*. Dev Biol 428(1):215-223.
Xu, S., and A. D. Chisholm

2011 A Galpha(q)-Ca(2+) signaling pathway promotes actin-mediated epidermal wound closure in *C. elegans*. Curr Biol 21:1960-1967.

Zugasti, O., et al.

2014 Activation of a G protein-coupled receptor by its endogenous ligand triggers the innate immune response of *Caenorhabditis elegans*. Nature immunology 15(9):833-8.
